# ARDebug: An Augmented Reality Tool for Analysing and Debugging Swarm Robotic Systems

**DOI:** 10.3389/frobt.2018.00087

**Published:** 2018-07-24

**Authors:** Alan G. Millard, Richard Redpath, Alistair M. Jewers, Charlotte Arndt, Russell Joyce, James A. Hilder, Liam J. McDaid, David M. Halliday

**Affiliations:** ^1^York Robotics Laboratory, University of York, York, United Kingdom; ^2^Department of Electronic Engineering, University of York, York, United Kingdom; ^3^Department of Computer Science, University of York, York, United Kingdom; ^4^School of Computing and Intelligent Systems, Ulster University, Derry, United Kingdom

**Keywords:** swarm robotics, augmented reality, debugging, open-source, cross-platform, code:c++

## Abstract

Despite growing interest in collective robotics over the past few years, analysing and debugging the behaviour of swarm robotic systems remains a challenge due to the lack of appropriate tools. We present a solution to this problem—ARDebug: an open-source, cross-platform, and modular tool that allows the user to visualise the internal state of a robot swarm using graphical augmented reality techniques. In this paper we describe the key features of the software, the hardware required to support it, its implementation, and usage examples. ARDebug is specifically designed with adoption by other institutions in mind, and aims to provide an extensible tool that other researchers can easily integrate with their own experimental infrastructure.

## 1. Introduction and related work

Debugging robotic systems is an inherently complex task, and the traditional software debugging tools currently available are of limited use. Erroneous behaviour can be attributed to either bugs in a robot's control code, or faults in its hardware, and determining the cause of the problem is often challenging due to the lack of feedback regarding the robot's internal state. Simple hardware such as on-board LEDs or LCD screens may be used to signal data to an experimenter (McLurkin et al., 2006), however such methods are only able to convey small quantities of information. Remotely connecting to a robot may allow an experimenter to capture detailed debug messages, but this kind of output can be difficult to correlate with the behaviour of the robot in real-time.

These problems are exacerbated when working with swarm robotic systems (Brambilla et al., 2013), where the quantity of debugging information required to locate and resolve a bug increases with the number of robots in the system. Large volumes of text-based debug messages concurrently transmitted by multiple robots are difficult for a human experimenter to interpret in real-time, and can easily become overwhelming. Moreover, the behaviour of an individual robot in a swarm robotic system is a product of not only its hardware, software, and interactions with its environment, but also its interactions with other robots. This increases the number of variables that may affect a specific robot's behaviour, which an experimenter must keep track of in order to isolate a bug.

Various debugging tools already exist for single- and multi-robot systems, but very few have been created specifically for use with robot swarms. Perhaps the most general-purpose tools are the rviz^1^ 3D visualiser and rqt^2^ GUI development framework for the Robot Operating System (ROS) (Quigley et al., 2009). However, due to its centralised architecture, ROS has not yet been widely adopted by the swarm robotics community. Following the recent official release of ROS 2.0, which features a distributed architecture, ROS may eventually gain traction with swarm roboticists. Until then, new tools and techniques are required to make the task of analysing and debugging the behaviour of swarm robotic systems easier for researchers.

It has been shown that *augmented reality*—the process of superimposing computer-generated graphics onto a view of the real world, to create a composite perspective (Azuma, 1997)—can be used to overcome some of the limitations of traditional debugging tools when engineering robotic systems (Collett and MacDonald, 2010). For example, Ghiringhelli et al. (2014) demonstrated a promising approach to debugging multi-robot systems, by augmenting a video feed with real-time information obtained from the robots being observed. Although the generalisability of their system was somewhat limited due to dependence on specialised robotic hardware, they showed that augmented reality tools can be used effectively for debugging multi-robot systems.

This paper presents ARDebug—a novel augmented reality tool that is specifically designed for analysing and debugging the behaviour of swarm robotic systems, which builds on the success of Ghiringhelli et al. (2014), offering a more generalised tool that can meet the requirements of a wider variety of swarm systems. It provides an experimenter with a single intuitive interface through which they can view the internal state of robots in a swarm in real-time, making the process of identifying, locating, and fixing bugs significantly easier. Similar *mixed reality* (Hoenig et al., 2015) applications exist that allow robots to perceive augmented reality environments through the use of virtual sensors (Reina et al., 2015, 2017; Antoun et al., 2016), which can aid the debugging process through the creation of reproducible virtual environments, but ARDebug differs from these tools in its focus on presenting detailed debugging information to a human experimenter.

## 2. System architecture

ARDebug works in real-time, tracking each of the robots within a video feed and combining their position information with other internal data obtained wirelessly from the robots. By fusing these data sources, researchers are provided with a new tool for identifying bugs and diagnosing faults in robot swarms. Users are able to compare the internal data that defines each robot's “understanding” of its environment, against a view of that same environment, thus making any perception anomalies apparent.

An experimenter is also able to observe the behaviour of a swarm robotic system while simultaneously being informed of changes to each robot's state. ARDebug uses graphical augmented reality techniques alongside more traditional textual/visual data presentation to make this possible, with the aim of reducing the time required for a user to identify bugs or faults. The primary use case for the system is in lab-based development and debugging of new robot behaviours, prior to their use in experimental work or deployment in the field.

The experimental infrastructure required for the debugging system presented in this paper comprises three main components: the robots themselves, a tracking system, and the core ARDebug software (described in section 3). A host machine collects data sent wirelessly from the individual robots, as well as position and orientation (or *pose*) data from a tracking system. The application then combines these sources of information and presents them to the user.

### 2.1. Tracking infrastructure

In our own experimental setup, the position and orientation of each robot is tracked using a JAI GO 5000C-PGE 5-megapixel camera (maximum resolution of 2,560 × 2,048), and the ArUco fiducial marker detection library (Garrido-Jurado et al., 2014). The camera is positioned 2.5 m above the centre of the arena, which is approximately 2.5 m square. A 5.2 cm square ArUco marker, is attached to the top of each robot and tracked by the camera using ArUco image processing algorithms built into OpenCV (Bradski, 2000). The image coordinates of each tag can be easily converted into real-world coordinates using a simple camera back-projection model (Sturm et al., 2011).

The use of a passive marker-based tracking algorithm means that no additional hardware is required to track the robots; any robot can be tracked as long as a fiducial marker can be attached to it. The minimum tracking hardware required to use ARDebug is incredibly low cost—ArUco tags that can simply be printed on paper, and a single visible-light camera of sufficient resolution to decode the fiducial markers at the desired distance. Inexpensive low-resolution cameras can be used if necessary, either by printing larger ArUco tags, or by generating a set of markers that are easier to distinguish at longer distances.

Alternative tracking systems can also be integrated into the system with relative ease, by sending tracking data to the core ARDebug application in JSON^3^ format. For example, an infra-red (IR) motion capture system such as OptiTrack (Millard et al., 2014) or Vicon^4^ could be used instead in conjunction with a visible-light camera.

### 2.2. Robot platform

ARDebug is designed to be agnostic to the robot platform used—any robot that can transmit its internal state in JSON format via Wi-Fi or Bluetooth can be integrated with the software. We provide example code for integrating the system with two different swarm robotic platforms: the widely-used e-puck (Mondada et al., 2009) via Wi-Fi, and the Psi Swarm (Hilder et al., 2016) via Bluetooth.

In order to enable Wi-Fi communication with ARDebug, we have equipped each of our e-puck robots with a Linux extension board (Liu and Winfield, 2011) featuring an ARM processor and a Wi-Fi adapter. Robot control code was written for the ARM processor using the ARGoS framework (Garattoni et al., 2015), which communicates with the e-puck's dsPIC microcontroller that interfaces with the robot's sensors and actuators. This extension board could equivalently be replaced with the popular Gumstix Overo COM turret^5^, or the new Pi-puck extension board (Millard et al., 2017), which extends the e-puck's capabilities by interfacing it with a Raspberry Pi single-board computer.

The Psi Swarm robot instead communicates with ARDebug via Bluetooth, using control code written for its mbed microcontroller. Despite using a different communication protocol, the data transmitted to/from the robot conforms to the same JSON interface. These two sets of example code are provided to make it easy for other users to integrate their own robot platforms with ARDebug.

## 3. ARdebug software

ARDebug displays data retrieved from the robots superimposed onto a video feed of the arena, in conjunction with robot pose data obtained via a tracking system. The design of the user interface focuses on presenting information to the user in a structured manner that is readily understood. High-level information is made available by means of graphical and textual overlays, with the ability to access more detailed information about the selected robot(s) via data tables and real-time charts. Previous research into interfaces for interacting with multi-robot systems has shown that users prefer this type of design (Rule and Forlizzi, 2012). Due to the volumes of data present in swarm systems, these filtering capabilities are critically important for focusing on the key data elements relevant to the task at hand.

### 3.1. Key features

**Figure 1:**
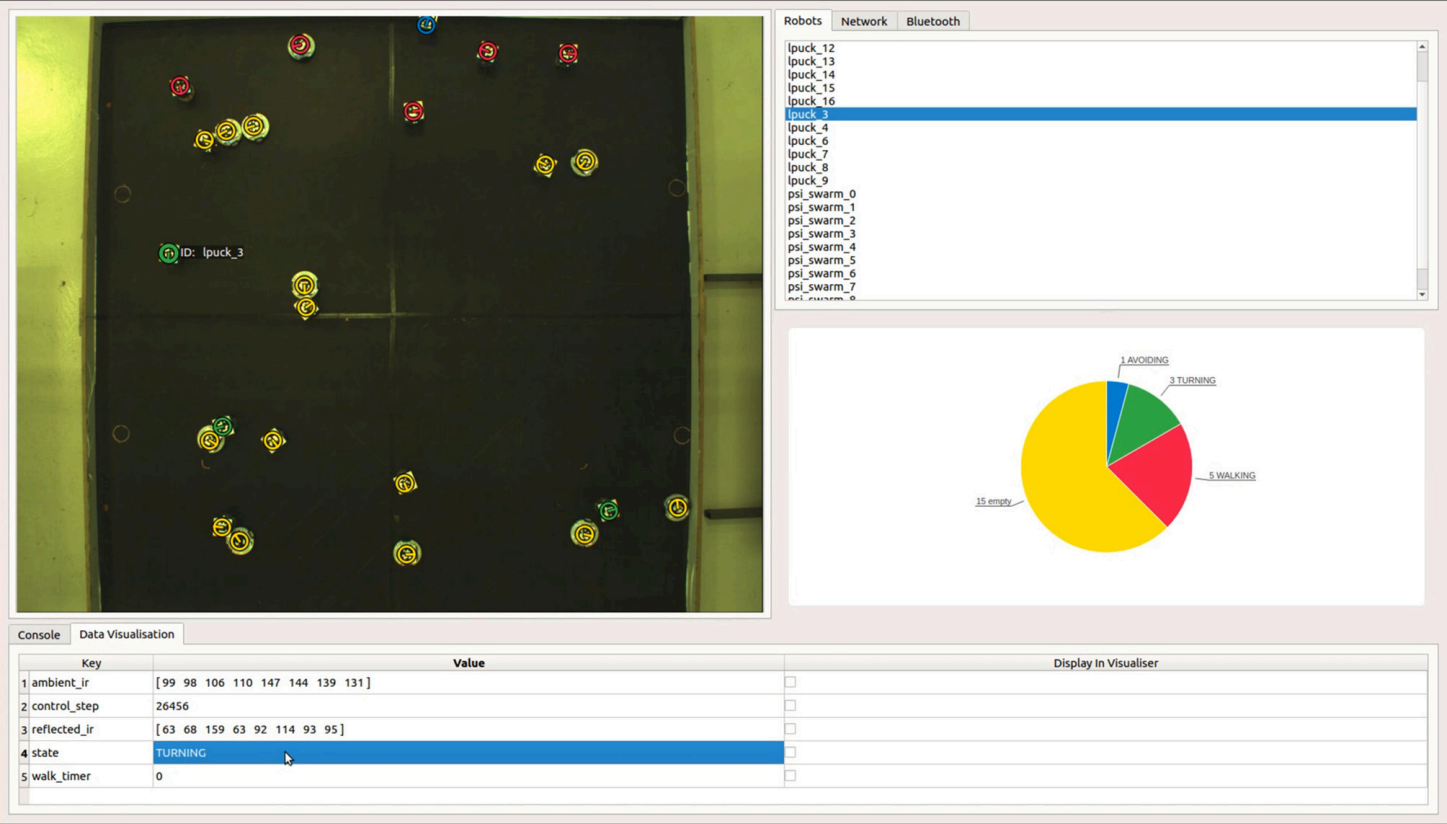
The ARDebug user interface, showing a top-down view of a heterogeneous swarm comprising Linux-enhanced e-pucks and Psi Swarm robots. The *Data Visualisation* tab shows internal data received from the selected robot that can optionally be overlaid onto the video feed, along with the robot's recent trajectory history and graphical representations of its sensor readings. In this particular example, a real-time pie chart is also used to visualise the distribution of internal state data across the swarm.

The ARDebug GUI, shown in Figure 1, is split into four regions. The visualiser (top-left) displays the augmented video feed, and can generate overlays showing a robot's position, orientation, ID, and any data that the robot reports via the JSON interface. Each of these overlays can be individually set to display for all of the selected robots in the system, or for only one robot. The connection pane (top-right), is used to display a list of the robots currently known to the system, and tabs allow access to network controls, Bluetooth device management, and data logging settings. The details pane (bottom) is used to display data transmitted by the selected robot(s), which can be overlaid onto the video feed, or displayed visually as a real-time chart in the fourth region of the application. A video demonstrating the use of these features to debug a swarm robotic system can be found on our website^6^.

The *Data Visualisation* tab lists data transmitted by the selected robot(s) to the ARDebug application. This data is formatted as a series of key/value pairs, allowing the user to report any information from within their robot code that they deem important to the debugging process. For example, the user can define custom data fields such as the robot's current battery voltage, control code iteration, or random walk timer value. Each of these custom data elements can be simultaneously displayed on the visualiser; battery voltage could even be rendered as a floating bar next to each robot if desired. Users can easily add their own visualisations to the software by subclassing an abstract visualisation element class, and drawing with geometric primitives.

These key/value pairs can also be visualised using real-time charts. For example, ARDebug will display any array of numerical values as a bar chart. This feature can be used to graphically represent a robot's sensor readings, such as ambient and reflected infra-red light. Strings, such as the robot's current state, are instead displayed as a pie chart showing the distribution of values across the selected robots, which are assigned colours in the visualiser in relation to the segments of the chart (as shown in Figure 1). This information can be useful to determine whether a robot is getting stuck in a particular state under certain conditions. Finally, single numerical values, such as a robot's battery voltage, are visualised as a line chart displaying the recent history of reported values over time.

ARDebug also offers a number of convenience features, including: data logging for post-experiment analysis; selection of robots via the visualiser, so they can be more easily differentiated or flagged for analysis; and the inclusion of ArUco tag detection as a core feature of the application, making it easier for users to get started with the software. The mapping between ArUco tags and robot IDs can be configured by simply modifying a JSON configuration file (a default mapping file is generated if one is not found).

### 3.2. Implementation

**Figure 2:**
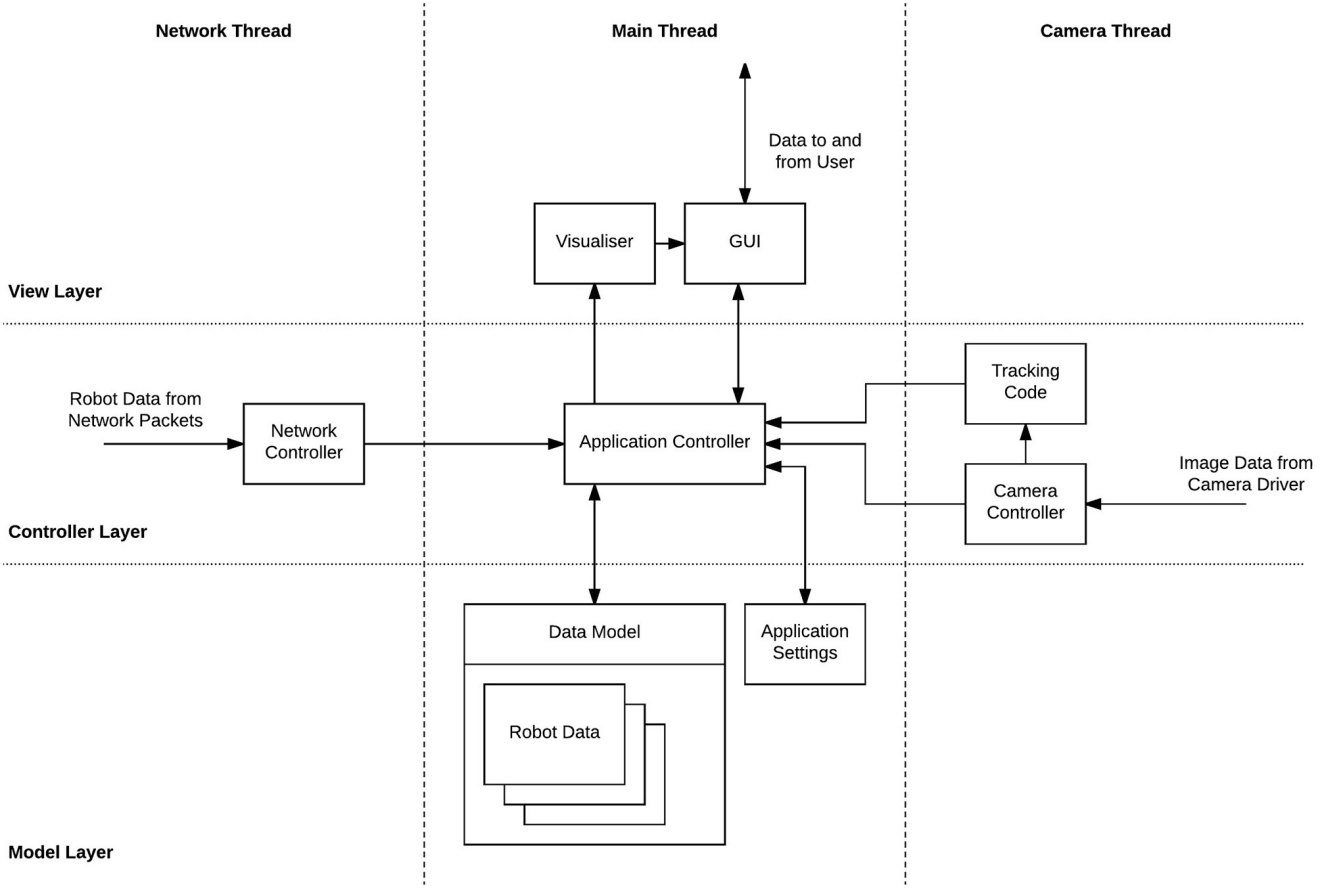
ARDebug software architecture. Components are split across Model, View, and Controller layers, and Main, Network, and Camera threads.

ARDebug has been implemented following a model-view-controller (MVC) architecture (Krasner and Pope, 1988), and is designed to be highly modular. Figure 2 shows a breakdown of the software architecture, including the key modules, organised according to both MVC layer and threading.

The *Model* layer contains data describing the current state of the robots, which is dealt with by the *data model*. New data from the robots or tracking system is received and parsed in the *Controller* layer, on task-specific threads, before being passed to the *application controller*, which handles updating the model. The *View* layer contains the user interface and is updated regularly to display the contents of the model, as well as receiving and passing user input messages to the central application controller. Using an MVC architecture in this way ensures that only one copy of the data describing the robots is maintained, and state information is not entangled within the user interface. Combined with the modular design, this allows individual elements of the software to be easily replaced without disrupting other elements or the overall structure.

The software is currently known to work under Ubuntu 16.04 or later, and macOS 10.13.5. OpenCV and the Qt application framework^7^ were chosen as the base upon which to build the ARDebug software, as they are mature, cross-platform libraries, with refined APIs and extensive documentation. They are also free, open-source software—Qt is released under GNU Lesser General Public License v3.0; and OpenCV, along with its implementation of the ArUco library, is released under the 3-Clause BSD License. It is hoped that the use of these libraries will enhance the maintainability and longevity of ARDebug.

### 3.3. Scalability

We have tested ARDebug with heterogeneous swarms of up 25 robots (15 e-pucks and 10 Psi Swarm robots), communicating with the application via a combination of Wi-Fi and Bluetooth in 100 ms intervals. With the software running on a server housing a 16-core Intel Xeon E5520 (2.27 GHz) and 16 GB RAM, ARDebug was able to track all of the robots and visualise their internal state in real-time. OpenCV's ArUco library implementation supports multi-threaded execution, so scales well as the number of robots in a swarm increases. We have verified that the software is able to track up to 50 ArUco tags in real-time, which is representative of the number of robots used in large-scale swarm experiments [excluding miniature robotic platforms such as Kilobots (Rubenstein et al., 2012) or Droplets (Farrow et al., 2014), which are not supported by ARDebug].

If the experimental arena covers a large spatial area, then it may be necessary to stitch together images from multiple overhead cameras in order to track an entire swarm. If high resolution cameras are used, the computational load can be distributed by performing ArUco tag detection on multiple servers, which then send tracking data to the ARDebug application via the JSON interface.

The performance of the application (post-tracking) is largely independent of number of robots, the amount of data they send, and the frequency they send it at. This is because ARDebug collates received data over a short time window before triggering an update to the GUI, instead of updating the user interface upon receipt of each JSON packet.

### 3.4. Current limitations

ARDebug is currently limited to tracking robots in a 2D plane, but could be extended for use with flying robots moving in three dimensions if integrated with a tracking system such as OptiTrack or Vicon. The application is also only able to integrate with robotic platforms that can communicate with a server via Bluetooth or Wi-Fi. This therefore excludes Kilobots (Rubenstein et al., 2012) and Droplets (Farrow et al., 2014), which are only able to communicate via IR light.

An inherent limitation of the Bluetooth specification means that only 7 robots can simultaneously connect to a single Bluetooth adapter. In order to use ARDebug with more than 7 Bluetooth-connected robots, it is necessary to use multiple adapters that communicate with up to 7 robots each. If Wi-Fi/Bluetooth communication is used for inter-robot communication as part of an experiment, then also sending data to ARDebug may create a communication bottleneck. However, this can be mitigated by reducing the amount and frequency of data transmitted to the application.

## 4. Conclusions

Appropriate debugging tools are necessary if the behaviour of swarm robotic systems are to be analysed and debugged effectively. In particular, tools are needed that allow an experimenter to visually inspect and interpret the data held by each robot in the system. This paper has presented ARDebug—a tool for monitoring a robot swarm by displaying its internal data in real-time. Augmented reality techniques are employed to visualise the data, with the aim of making it readily understandable, and therefore quicker to parse than numerical or textual data alone.

ARDebug aims for minimal hardware requirements, cross-platform compatibility, and is implemented in a modular fashion to allow easy modification and integration with different hardware. The software is open-source (released under the GNU General Public License v3.0), and has been made freely available online in the hope that it will contribute a useful tool to the field of swarm robotics research: 10.5281/zenodo.1283488 (Datasheet 1 in Supplementary Material).

Scenarios in which ARDebug is envisioned to be useful include: diagnosing robot control code bugs, identifying sensor hardware faults or calibration issues, and verifying actuator hardware operation. Access to internal state information and decision-making variables could aid in debugging robot control code, for instance, by checking that state transitions are occurring in response to stimuli, or by checking that variables are being updated correctly.

A number of future extensions to the system are planned: support for scripting custom video augmentations, to allow users to more easily tailor the system to their own needs; video recording and data log replay, for more in-depth post-experiment analysis and debugging; and bidirectional communication between ARDebug and the robots, allowing the software to be used as a central experiment controller.

## Author contributions

AM, RR, and AJ wrote sections of the paper. AJ wrote the initial version of the software and documentation, which was then developed further by AM, RR, and CA. AM, RJ, and JH worked on experimental infrastructure that enabled the development of the software. LM and DH supervised the project.

### Conflict of interest statement

The authors declare that the research was conducted in the absence of any commercial or financial relationships that could be construed as a potential conflict of interest.

## Footnotes

^1^http://wiki.ros.org/rviz

^2^http://wiki.ros.org/rqt

^3^https://www.json.org

^4^https://www.vicon.com

^5^http://www.gctronic.com/doc/index.php/Overo_Extension

^6^https://www.york.ac.uk/robot-lab/ardebug/

^7^https://www.qt.io
