# Supplementary figures and images for: ARDebug: An Augmented Reality Tool for Analysing and Debugging Swarm Robotic Systems
Source: Front Robot AI. 2018 Jul 24;5:87. doi: 10.3389/frobt.2018.00087 (PMC7805932; doi:10.3389/frobt.2018.00087)

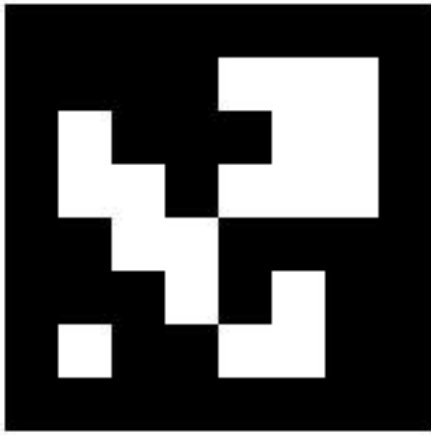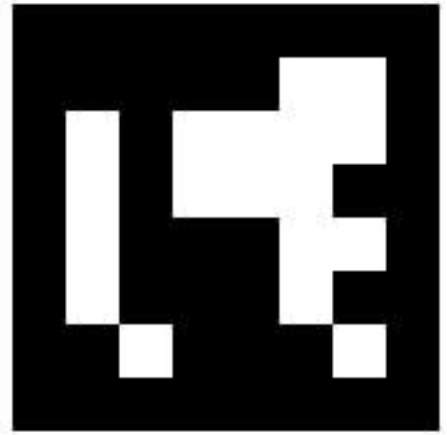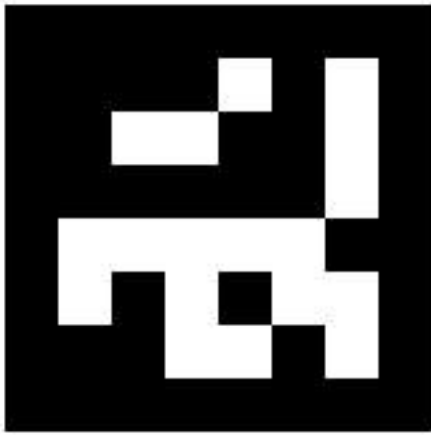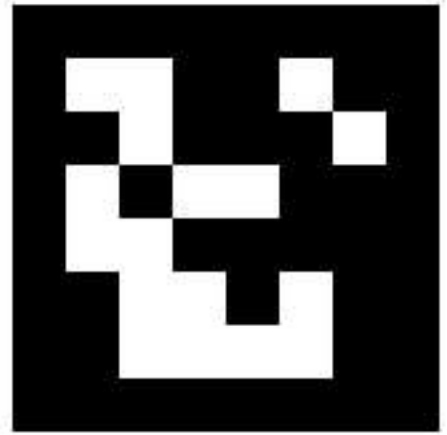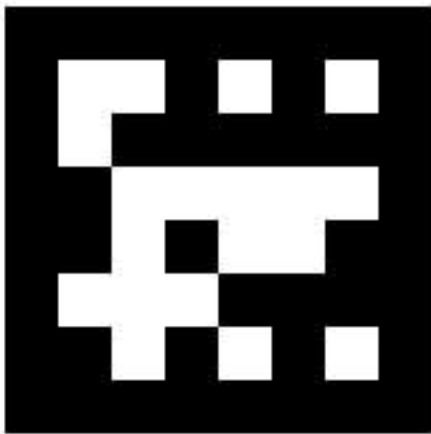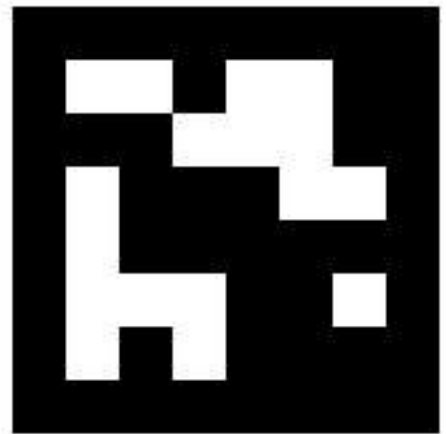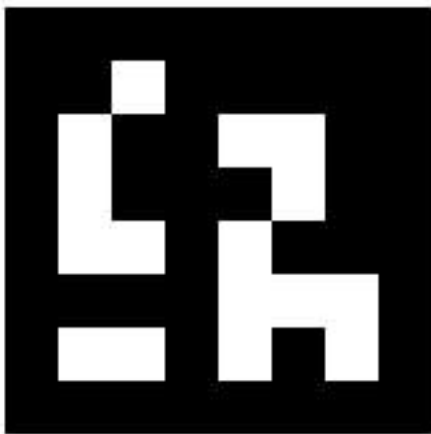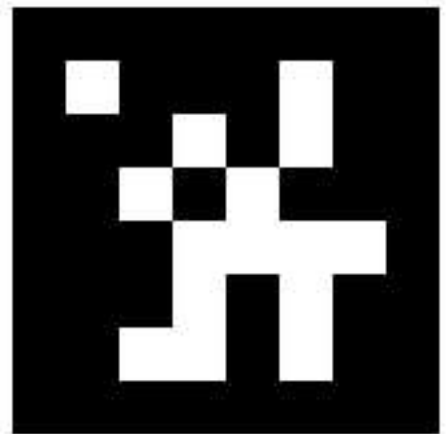

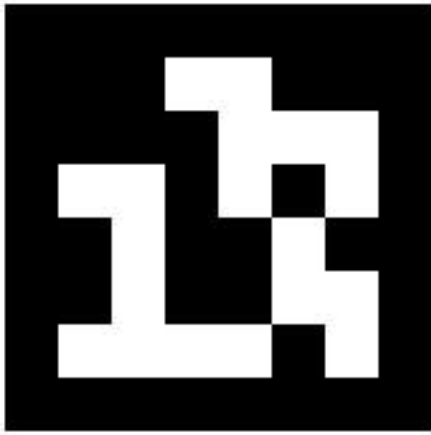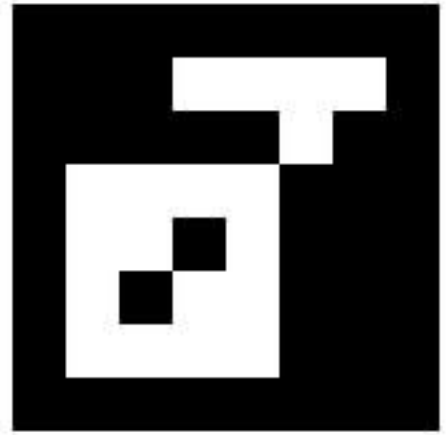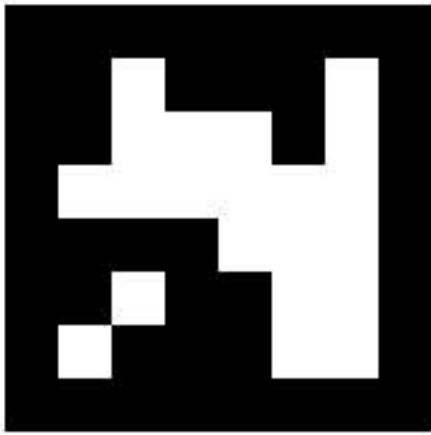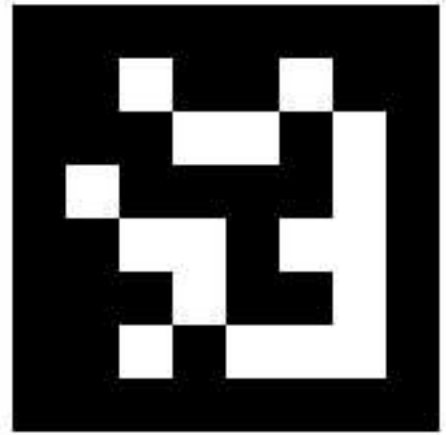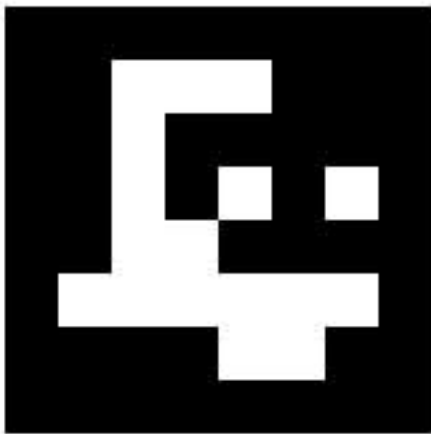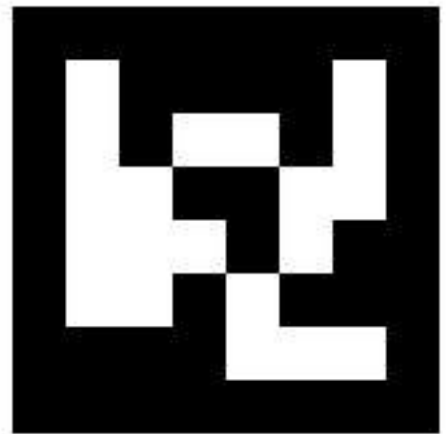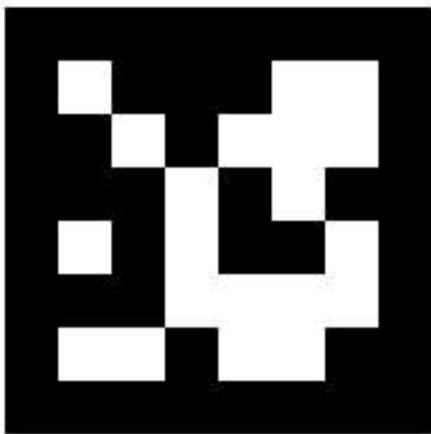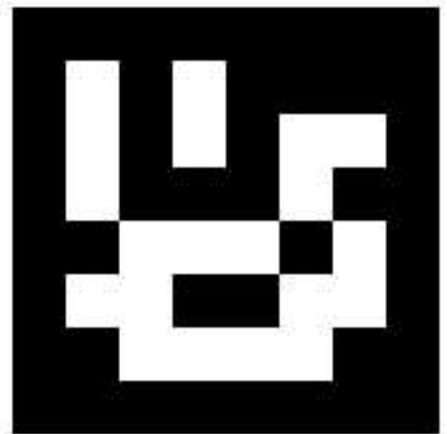

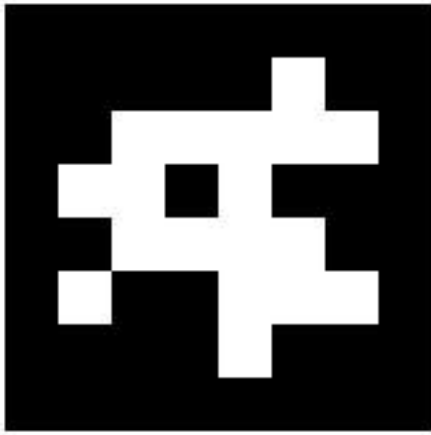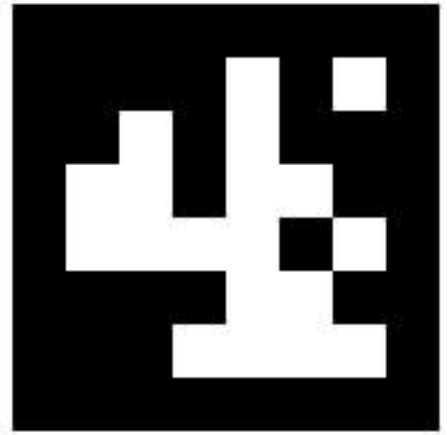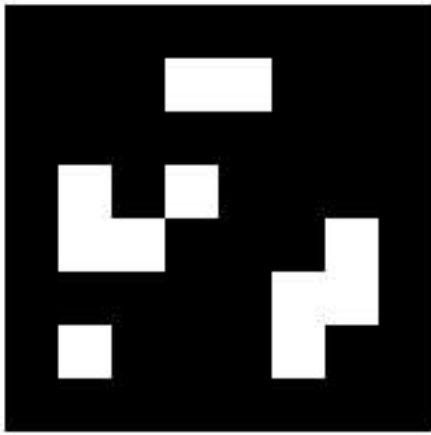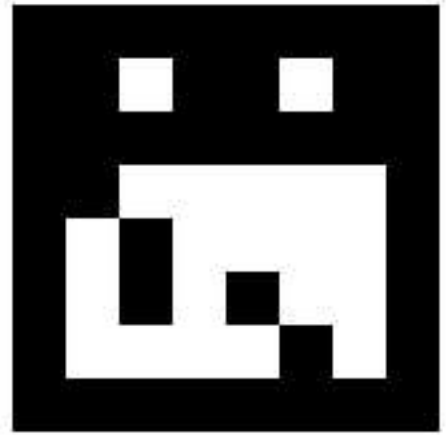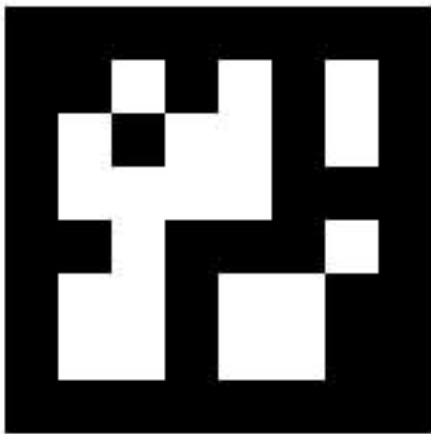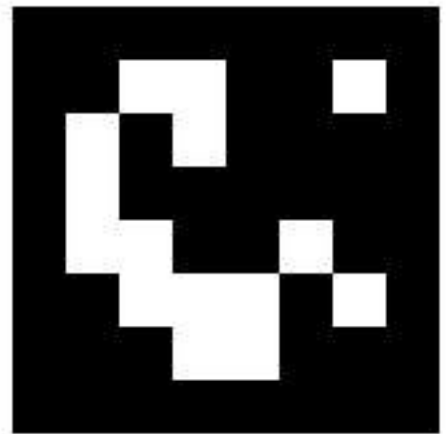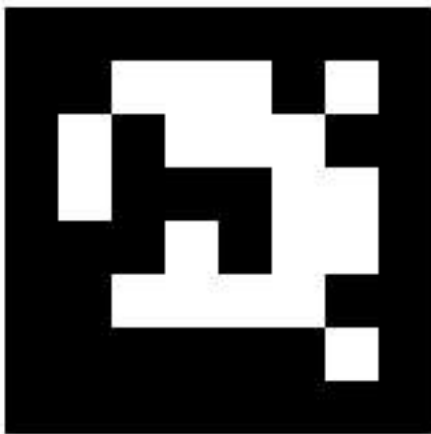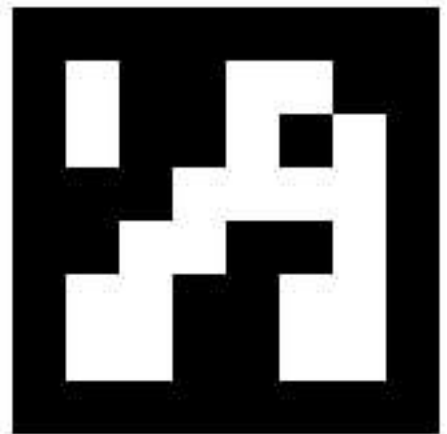

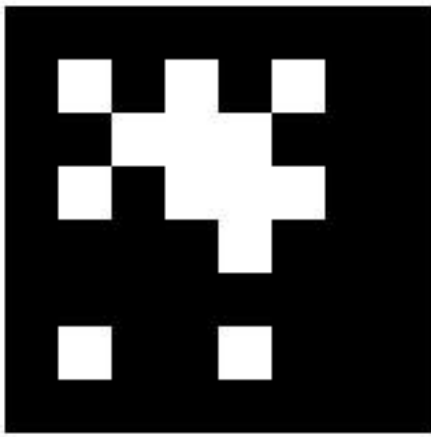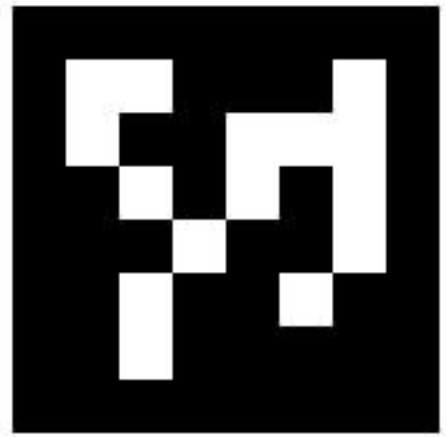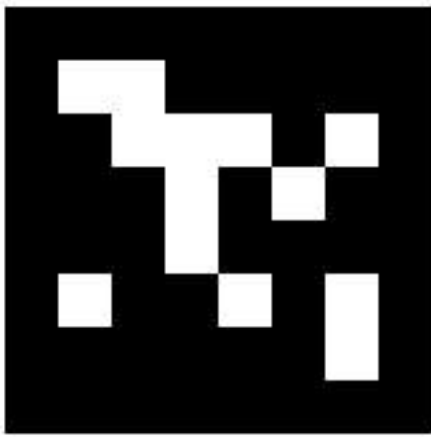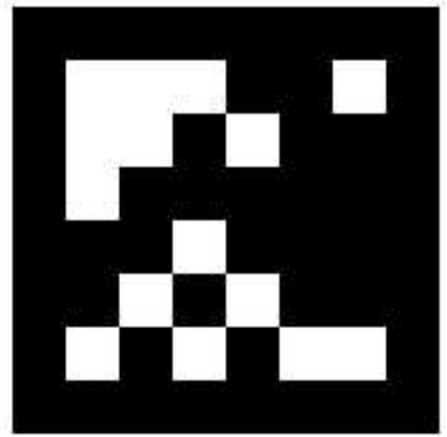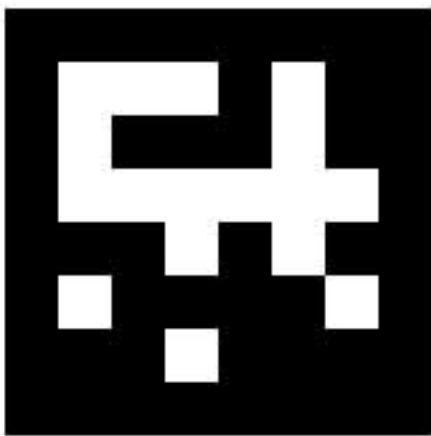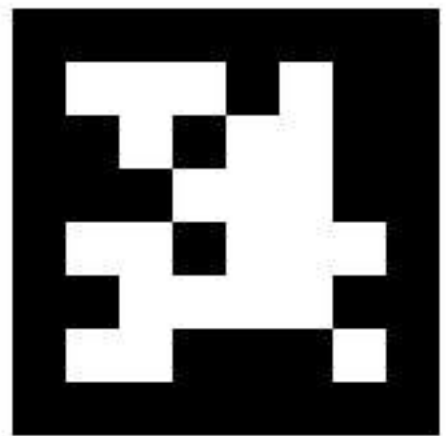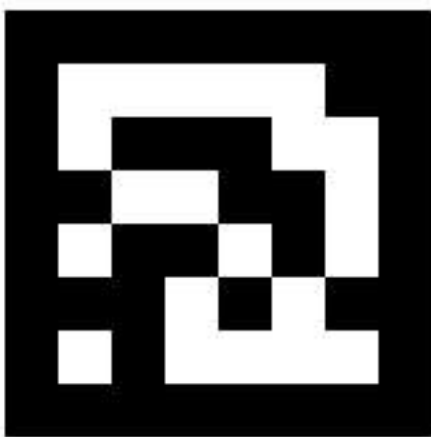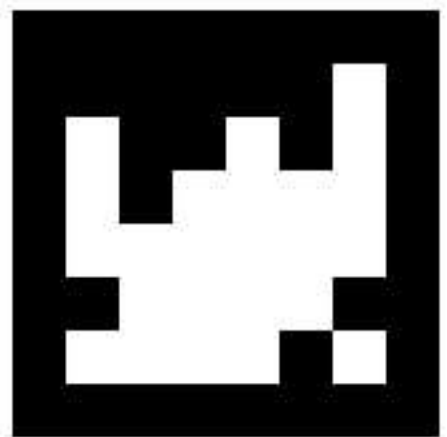

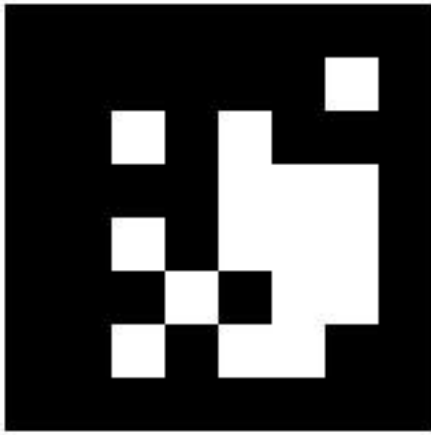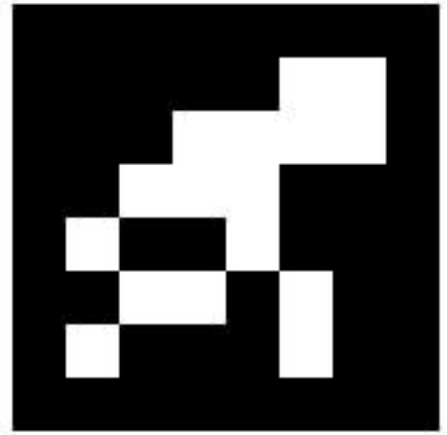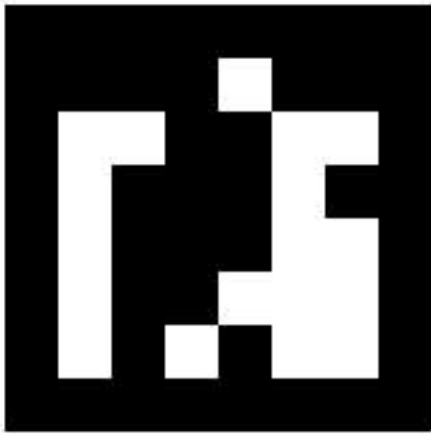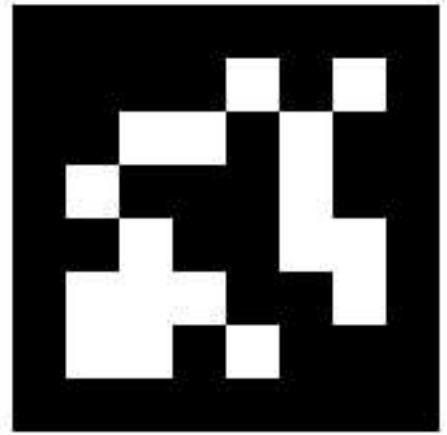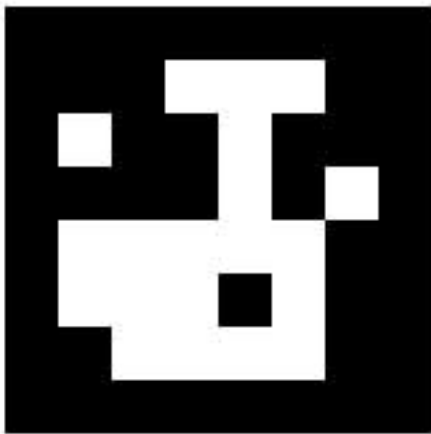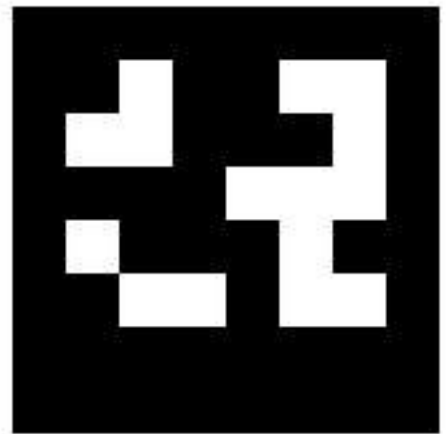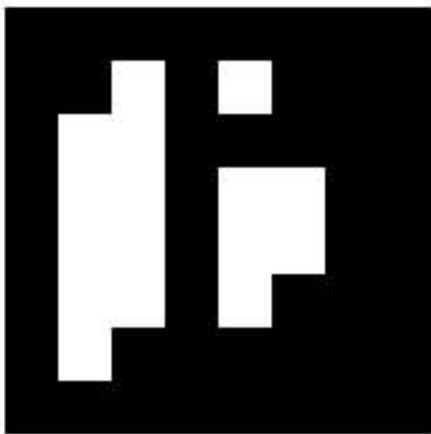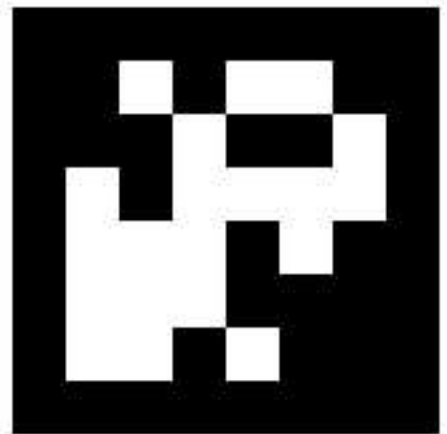

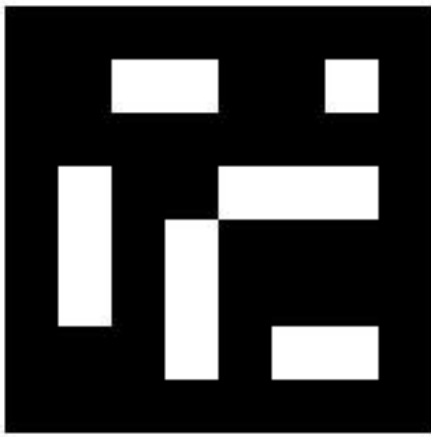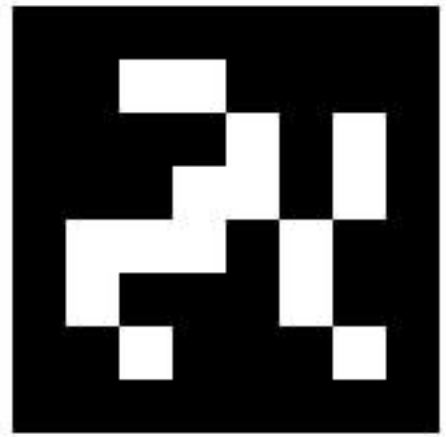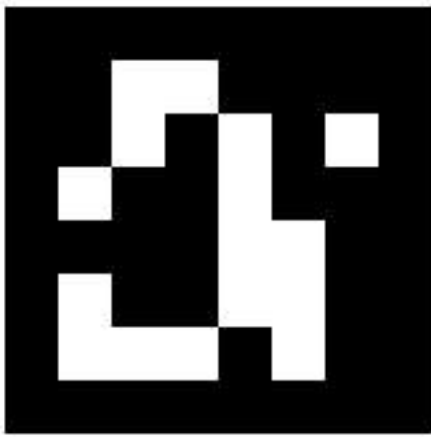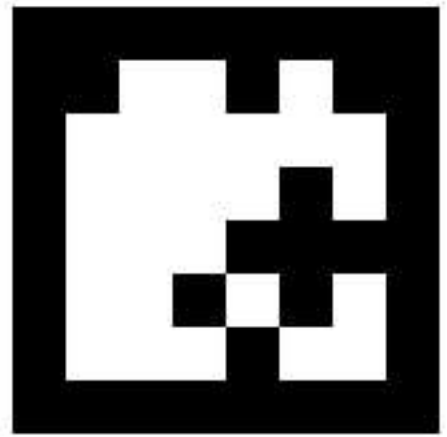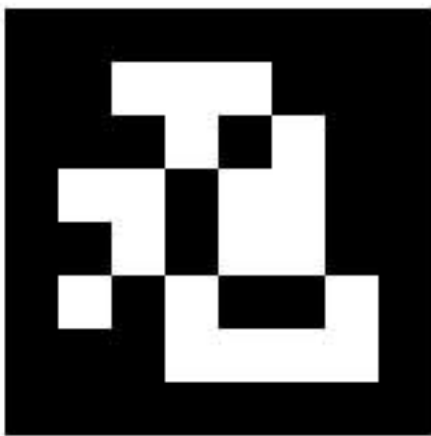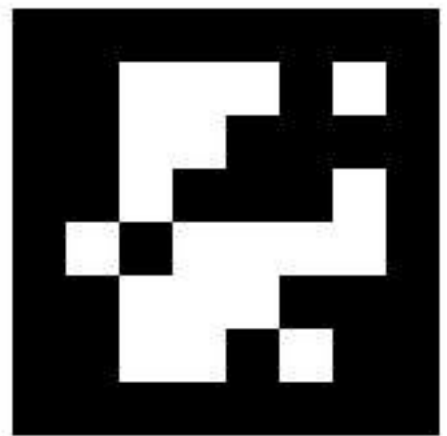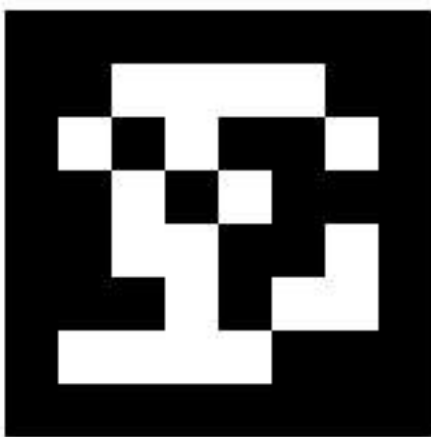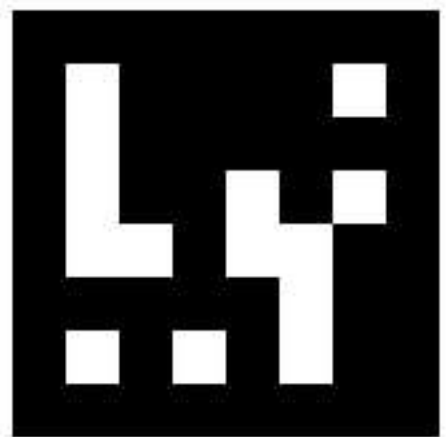

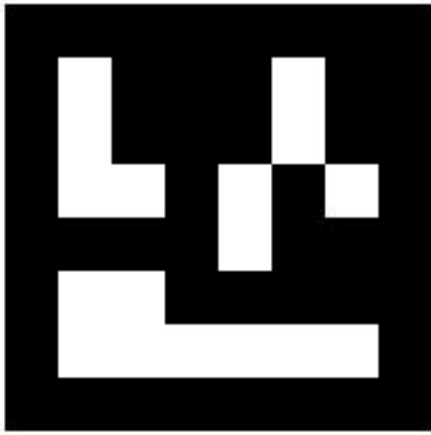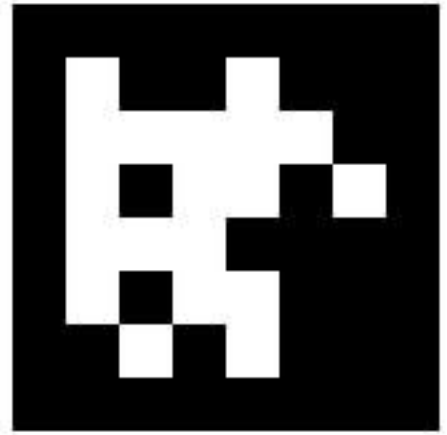

Supplement: Supplementary file 1 [file Data_Sheet_1.ZIP › yorkrobotlab-ardebug-93b24cd/ArUcoMarkers/allMarker.pdf]

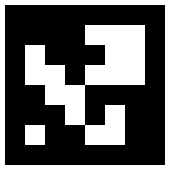

Supplement: Supplementary file 1 [file Data_Sheet_1.ZIP › yorkrobotlab-ardebug-93b24cd/ArUcoMarkers/marker_0.png]

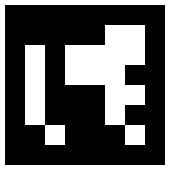

Supplement: Supplementary file 1 [file Data_Sheet_1.ZIP › yorkrobotlab-ardebug-93b24cd/ArUcoMarkers/marker_1.png]

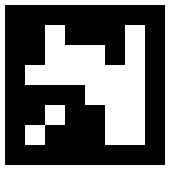

Supplement: Supplementary file 1 [file Data_Sheet_1.ZIP › yorkrobotlab-ardebug-93b24cd/ArUcoMarkers/marker_10.png]

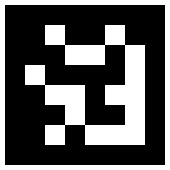

Supplement: Supplementary file 1 [file Data_Sheet_1.ZIP › yorkrobotlab-ardebug-93b24cd/ArUcoMarkers/marker_11.png]

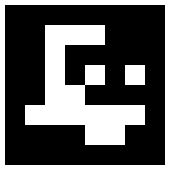

Supplement: Supplementary file 1 [file Data_Sheet_1.ZIP › yorkrobotlab-ardebug-93b24cd/ArUcoMarkers/marker_12.png]

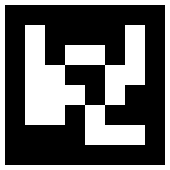

Supplement: Supplementary file 1 [file Data_Sheet_1.ZIP › yorkrobotlab-ardebug-93b24cd/ArUcoMarkers/marker_13.png]

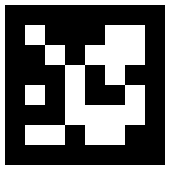

Supplement: Supplementary file 1 [file Data_Sheet_1.ZIP › yorkrobotlab-ardebug-93b24cd/ArUcoMarkers/marker_14.png]

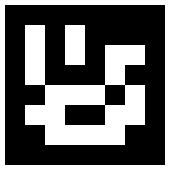

Supplement: Supplementary file 1 [file Data_Sheet_1.ZIP › yorkrobotlab-ardebug-93b24cd/ArUcoMarkers/marker_15.png]

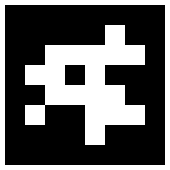

Supplement: Supplementary file 1 [file Data_Sheet_1.ZIP › yorkrobotlab-ardebug-93b24cd/ArUcoMarkers/marker_16.png]

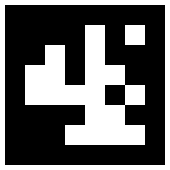

Supplement: Supplementary file 1 [file Data_Sheet_1.ZIP › yorkrobotlab-ardebug-93b24cd/ArUcoMarkers/marker_17.png]

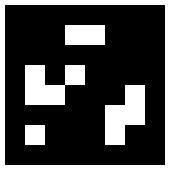

Supplement: Supplementary file 1 [file Data_Sheet_1.ZIP › yorkrobotlab-ardebug-93b24cd/ArUcoMarkers/marker_18.png]

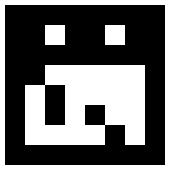

Supplement: Supplementary file 1 [file Data_Sheet_1.ZIP › yorkrobotlab-ardebug-93b24cd/ArUcoMarkers/marker_19.png]

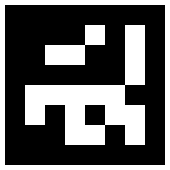

Supplement: Supplementary file 1 [file Data_Sheet_1.ZIP › yorkrobotlab-ardebug-93b24cd/ArUcoMarkers/marker_2.png]

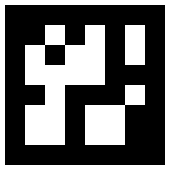

Supplement: Supplementary file 1 [file Data_Sheet_1.ZIP › yorkrobotlab-ardebug-93b24cd/ArUcoMarkers/marker_20.png]

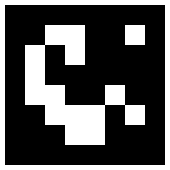

Supplement: Supplementary file 1 [file Data_Sheet_1.ZIP › yorkrobotlab-ardebug-93b24cd/ArUcoMarkers/marker_21.png]

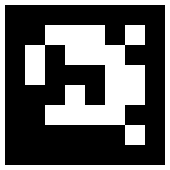

Supplement: Supplementary file 1 [file Data_Sheet_1.ZIP › yorkrobotlab-ardebug-93b24cd/ArUcoMarkers/marker_22.png]

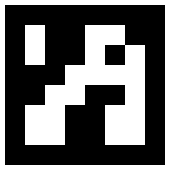

Supplement: Supplementary file 1 [file Data_Sheet_1.ZIP › yorkrobotlab-ardebug-93b24cd/ArUcoMarkers/marker_23.png]

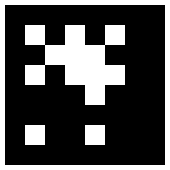

Supplement: Supplementary file 1 [file Data_Sheet_1.ZIP › yorkrobotlab-ardebug-93b24cd/ArUcoMarkers/marker_24.png]

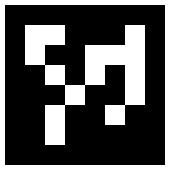

Supplement: Supplementary file 1 [file Data_Sheet_1.ZIP › yorkrobotlab-ardebug-93b24cd/ArUcoMarkers/marker_25.png]

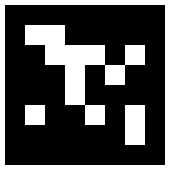

Supplement: Supplementary file 1 [file Data_Sheet_1.ZIP › yorkrobotlab-ardebug-93b24cd/ArUcoMarkers/marker_26.png]

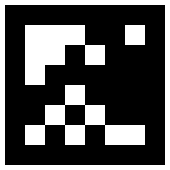

Supplement: Supplementary file 1 [file Data_Sheet_1.ZIP › yorkrobotlab-ardebug-93b24cd/ArUcoMarkers/marker_27.png]

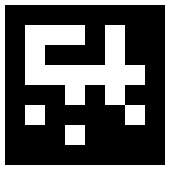

Supplement: Supplementary file 1 [file Data_Sheet_1.ZIP › yorkrobotlab-ardebug-93b24cd/ArUcoMarkers/marker_28.png]

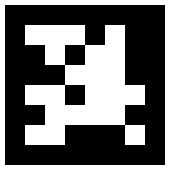

Supplement: Supplementary file 1 [file Data_Sheet_1.ZIP › yorkrobotlab-ardebug-93b24cd/ArUcoMarkers/marker_29.png]

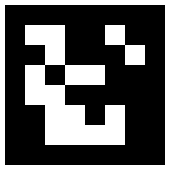

Supplement: Supplementary file 1 [file Data_Sheet_1.ZIP › yorkrobotlab-ardebug-93b24cd/ArUcoMarkers/marker_3.png]

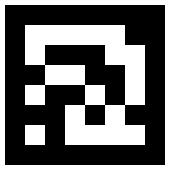

Supplement: Supplementary file 1 [file Data_Sheet_1.ZIP › yorkrobotlab-ardebug-93b24cd/ArUcoMarkers/marker_30.png]

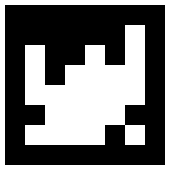

Supplement: Supplementary file 1 [file Data_Sheet_1.ZIP › yorkrobotlab-ardebug-93b24cd/ArUcoMarkers/marker_31.png]

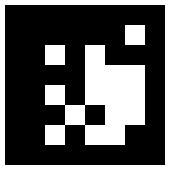

Supplement: Supplementary file 1 [file Data_Sheet_1.ZIP › yorkrobotlab-ardebug-93b24cd/ArUcoMarkers/marker_32.png]

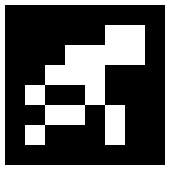

Supplement: Supplementary file 1 [file Data_Sheet_1.ZIP › yorkrobotlab-ardebug-93b24cd/ArUcoMarkers/marker_33.png]

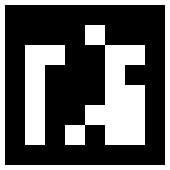

Supplement: Supplementary file 1 [file Data_Sheet_1.ZIP › yorkrobotlab-ardebug-93b24cd/ArUcoMarkers/marker_34.png]

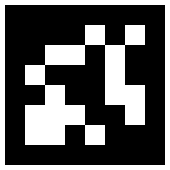

Supplement: Supplementary file 1 [file Data_Sheet_1.ZIP › yorkrobotlab-ardebug-93b24cd/ArUcoMarkers/marker_35.png]

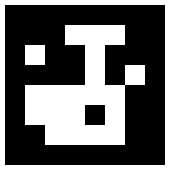

Supplement: Supplementary file 1 [file Data_Sheet_1.ZIP › yorkrobotlab-ardebug-93b24cd/ArUcoMarkers/marker_36.png]

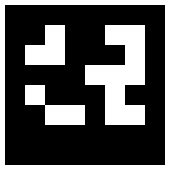

Supplement: Supplementary file 1 [file Data_Sheet_1.ZIP › yorkrobotlab-ardebug-93b24cd/ArUcoMarkers/marker_37.png]

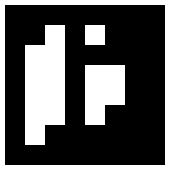

Supplement: Supplementary file 1 [file Data_Sheet_1.ZIP › yorkrobotlab-ardebug-93b24cd/ArUcoMarkers/marker_38.png]

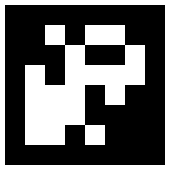

Supplement: Supplementary file 1 [file Data_Sheet_1.ZIP › yorkrobotlab-ardebug-93b24cd/ArUcoMarkers/marker_39.png]

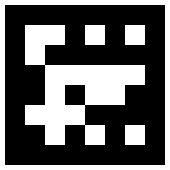

Supplement: Supplementary file 1 [file Data_Sheet_1.ZIP › yorkrobotlab-ardebug-93b24cd/ArUcoMarkers/marker_4.png]

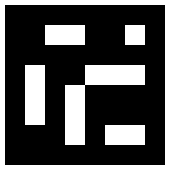

Supplement: Supplementary file 1 [file Data_Sheet_1.ZIP › yorkrobotlab-ardebug-93b24cd/ArUcoMarkers/marker_40.png]

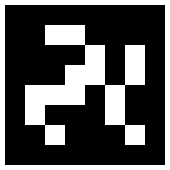

Supplement: Supplementary file 1 [file Data_Sheet_1.ZIP › yorkrobotlab-ardebug-93b24cd/ArUcoMarkers/marker_41.png]

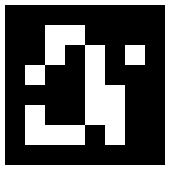

Supplement: Supplementary file 1 [file Data_Sheet_1.ZIP › yorkrobotlab-ardebug-93b24cd/ArUcoMarkers/marker_42.png]

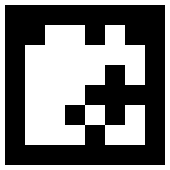

Supplement: Supplementary file 1 [file Data_Sheet_1.ZIP › yorkrobotlab-ardebug-93b24cd/ArUcoMarkers/marker_43.png]

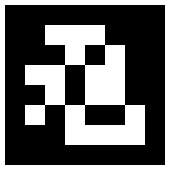

Supplement: Supplementary file 1 [file Data_Sheet_1.ZIP › yorkrobotlab-ardebug-93b24cd/ArUcoMarkers/marker_44.png]

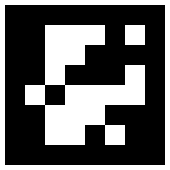

Supplement: Supplementary file 1 [file Data_Sheet_1.ZIP › yorkrobotlab-ardebug-93b24cd/ArUcoMarkers/marker_45.png]

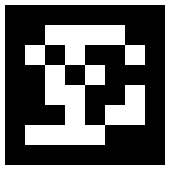

Supplement: Supplementary file 1 [file Data_Sheet_1.ZIP › yorkrobotlab-ardebug-93b24cd/ArUcoMarkers/marker_46.png]

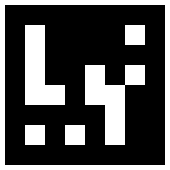

Supplement: Supplementary file 1 [file Data_Sheet_1.ZIP › yorkrobotlab-ardebug-93b24cd/ArUcoMarkers/marker_47.png]

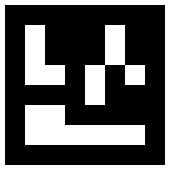

Supplement: Supplementary file 1 [file Data_Sheet_1.ZIP › yorkrobotlab-ardebug-93b24cd/ArUcoMarkers/marker_48.png]

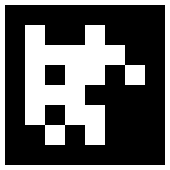

Supplement: Supplementary file 1 [file Data_Sheet_1.ZIP › yorkrobotlab-ardebug-93b24cd/ArUcoMarkers/marker_49.png]

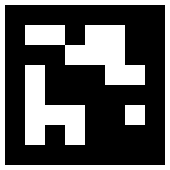

Supplement: Supplementary file 1 [file Data_Sheet_1.ZIP › yorkrobotlab-ardebug-93b24cd/ArUcoMarkers/marker_5.png]

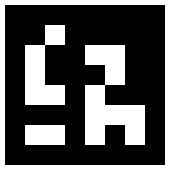

Supplement: Supplementary file 1 [file Data_Sheet_1.ZIP › yorkrobotlab-ardebug-93b24cd/ArUcoMarkers/marker_6.png]

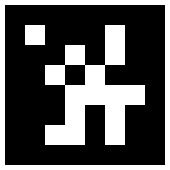

Supplement: Supplementary file 1 [file Data_Sheet_1.ZIP › yorkrobotlab-ardebug-93b24cd/ArUcoMarkers/marker_7.png]

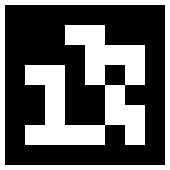

Supplement: Supplementary file 1 [file Data_Sheet_1.ZIP › yorkrobotlab-ardebug-93b24cd/ArUcoMarkers/marker_8.png]

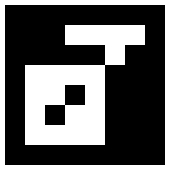

Supplement: Supplementary file 1 [file Data_Sheet_1.ZIP › yorkrobotlab-ardebug-93b24cd/ArUcoMarkers/marker_9.png]
